# Empfehlungen der DGRh-Kommission für Komplementäre Heilverfahren und Ernährung zur Anwendung von ausgewählten Phytotherapeutika und pflanzlichen Präparaten in der Rheumatologie

**DOI:** 10.1007/s00393-024-01612-w

**Published:** 2025-02-03

**Authors:** Gernot Keyßer, Olga Seifert, Inna Frohne, Andreas Michalsen, Alexander Pfeil, Monika Reuß-Borst, Oliver Sander

**Affiliations:** 1 Klinik und Poliklinik für Innere Medizin II, Universitätsklinikum Halle (Saale), Ernst-Grube-Str. 40, 06120 Halle (Saale), Deutschland; 2 Klinik und Poliklinik für Endokrinologie Nephrologie Rheumatologie, Universitätsklinikum Leipzig, 04103 Leipzig, Deutschland; 3 Privatpraxis für Rheumatologie, 45134 Essen, Deutschland; 4 Immanuel Krankenhaus, 14109 Berlin, Deutschland; 5 Klinik für Innere Medizin III, Universitätsklinikum Jena, 07747 Jena, Deutschland; 6 Facharztpraxis für Innere Medizin, 97708 Bad Bocklet, Deutschland; 7 Universitätsklinikum Düsseldorf, 40225 Düsseldorf, Deutschland

**Keywords:** Phytotherapie, Heilpflanzen, Rheumatische Erkrankungen, Degenerative Gelenkerkrankungen, Nahrungsergänzungsmittel, Phytotherapy, Medical plants, Rheumatic diseases, Degenerative joint diseases, Nutritional supplements

## Abstract

**Hintergrund:**

Pflanzliche Präparate und Phytotherapeutika werden für Symptome und Erkrankungen des rheumatischen Formenkreises angeboten und oft intensiv in der Laienpresse beworben. Die DGRh-Kommission für Komplementäre Heilverfahren und Ernährung hat die wissenschaftliche Literatur zu ausgewählten frei verkäuflichen Präparaten und von verschreibungsfähigen Phytotherapeutika gesichtet und Möglichkeiten für ihre Anwendung in der Rheumatologie geprüft.

**Methoden:**

In einer Online-Sitzung der Kommission am 08.02.2023 wurde eine Liste von pflanzlichen Präparaten erstellt, die in der Rheumatologie häufig Anwendung finden (meist als Selbstmedikation). Jedes Mitglied der Kommission führte daraufhin eine Literaturrecherche zu einer oder 2 Substanzen durch und fasste die Ergebnisse nach einer definierten Matrix zusammen. Dabei wurde zu Borretschöl, Brennnesselpräparaten, Cannabispräparaten sowie Zubereitungen von *Rosa canina*, Rosmarin, Safran und Weidenrinde recherchiert. Außerdem wurde die Datenlage zum Mischpräparat Phytodolor® (Bayer Vital GmbH, Deutschland) untersucht. Die Ergebnisse wurden im Umlaufverfahren überprüft und in 2 weiteren Online-Sitzungen der Kommission KHE konsentiert. Nach Prüfung durch den Vorstand der DGRh wurden die Empfehlungen auf die Website der Fachorganisation übernommen.

**Ergebnisse:**

Auch wenn für alle untersuchten Pflanzenstoffe Berichte über entzündungshemmende oder immunologische Effekte in vitro und/oder im Tiermodell vorliegen, ist die Evidenz für einen klinisch relevanten Nutzen in der Rheumatologie spärlich. Keines der untersuchten Präparate hat eine wissenschaftlich belegte therapeutische Wirksamkeit, die eine Anwendung bei entzündlichen Gelenkerkrankungen rechtfertigt. Präparate auf der Basis von Safran und Rosmarin werden generell nicht empfohlen. Borretschöl aus Samen kann bei standardisierter Herstellung im Rahmen einer gesundheitsbewussten Ernährung eingenommen werden, eine nennenswerte entzündungshemmende Wirkung ist nicht zu erwarten. Von Phytodolor® sowie Präparaten auf Basis von Brennnessel, Weidenrinde oder *Rosa canina*, die bei degenerativen Gelenkerkrankungen auf Patienteninitiative eingenommen werden, müssen Rheumatologen nicht abraten, wenn ansonsten ein sinnvolles Therapiekonzept eingehalten wird. Für medizinischen Cannabis existiert keine ausreichende Evidenz für eine Verordnung bei entzündlich rheumatischen Erkrankungen zur Krankheitsmodifikation oder zur symptomatischen Therapie. In Einzelfällen kann jedoch die Anwendung zur Reduktion von chronischen, insbesondere neuropathischen Schmerzen sowie Schlafstörungen und zur Reduktion des Opiatverbrauchs gerechtfertigt sein.

**Schlussfolgerung:**

Auch wenn die hier vorgestellten pflanzlichen Präparate für die rheumatologische Praxis differenziert betrachtet werden müssen, ist der Stellenwert der Phytotherapie für das Fachgebiet gering.

## Abstract (en)

**Background:**

Herbal preparations and phytotherapeutic substances are offered for symptoms and diseases of the rheumatic spectrum and are often intensively advertised in the lay press. The German Society for Rheumatology and Clinical Immunology (DGRh) Committee on Complementary Medicine and Nutrition reviewed the scientific literature on selected over the counter preparations and prescription phytotherapeutic substances and examined the possibilities for their use in rheumatology.

**Methods:**

In an online meeting of the Committee on 8 February 2023 a list of herbal preparations that are frequently used in rheumatology (mostly as self-medication) was drawn up. Each member of the committee then carried out a literature search on one or two substances and summarized the results according to a defined matrix. Research was carried out on borage oil, stinging nettle preparations, cannabis preparations and preparations of dog rose, rosemary, saffron and willow bark. The data on the mixed preparation Phytodolor® (Bayer Vital GmbH, Germany) were also examined. The results were reviewed by a circulation procedure and approved in two further online meetings of the Committee. After review by the DGRh board, the recommendations were transferred to the professional organization’s website.

**Results:**

Even though there are reports of anti-inflammatory or immunological effects in vitro and/or in animal models for all the plant substances examined, the evidence for a clinically relevant benefit is sparse. None of the preparations investigated has a therapeutic efficacy that justifies its use in inflammatory joint diseases. Herbal preparations based on saffron and rosemary are generally not recommended. Borage oil from seeds can be taken in standardized form as part of a health-conscious diet but is not expected to have any significant anti-inflammatory effect. Rheumatologists need not advise against Phytodolor® or preparations based on stinging nettle, willow bark or dog rose, which are taken on the patient’s initiative for degenerative joint diseases, if a sensible therapy concept is otherwise adhered to. There is insufficient evidence to prescribe medicinal cannabis for inflammatory rheumatic diseases for disease modification or symptomatic therapy. In individual cases, however, its use to reduce chronic pain, particularly neuropathic pain and sleep disorders and to reduce opiate consumption may be justified.

**Conclusion:**

Even if the herbal preparations presented here must be considered in a differentiated manner for rheumatology practice, the value of phytotherapy for the discipline is low.

## Einleitung

Pflanzliche Präparate zur Behandlung von rheumatischen Krankheitsbildern und muskuloskeletalen Symptomen finden unter Patienten und Ärzten große Beachtung. Auch in Zeiten spezifisch wirkender und gut verträglicher Basistherapien ist das wissenschaftliche Interesse an Phytotherapeutika und pflanzlichen Präparaten ungebrochen. Dies illustriert das Ergebnis einer Literaturrecherche in PubMed zu den Stichworten „herbal medicine“ und „arthritis“. Von den 4331 identifizierten Literaturstellen wurden über 1600 in den letzten 5 Jahren veröffentlicht (Stand 03.11.2024). Traditionell haben pflanzliche Präparate unter Betroffenen einen guten Leumund, und in der Taxonomie weisen viele deutsche und lateinische Pflanzennamen auf ihren Gebrauch als Heilpflanze hin: Neben dem Bergwohlverleih (*Arnica montana*) sei exemplarisch der Beinwell genannt (*Symphytum officinale*), der für Wundbehandlung eingesetzt wurde (altdeutsch „well“: das „Wohl“, griechisch „symphytein“: „zusammenwachsen“) oder die Osterluzei (*Aristolochia*), die in der Geburtshilfe Anwendung fand (griechisch „aristolocheía“, sinngemäß für „bestes Gebären“). Die Verwendung von pflanzlichen Heilmitteln beruht häufig auf empirischen Erfahrungen und Beobachtungen, in Teilen aber auch auf der frühchristlichen Signaturenlehre. Letztere postuliert, dass in Form und Farbe von Heilpflanzen göttliche Zeichen verborgen sind, die darauf hinweisen, für welche Krankheit sie therapeutisch genutzt werden können (z. B. Walnüsse für Erkrankungen des Gehirns). Ein maßgeblicher Verfechter dieser Lehre war Paracelsus [1]. Allerdings ist die therapeutische Potenz einer rein pflanzlichen Medizin begrenzt, und der Satz, dass gegen bestimmte Leiden „kein Kraut gewachsen“ ist, illustriert die Grenzen der Phytotherapie.

Für gesundheitliche Zwecke werden in Europa Phytopharmaka (Synonym: Phytotherapeutika), pflanzliche Präparate sowie Nahrungsergänzungsmittel (NEM) angewendet. Phytopharmaka haben eine reguläre Arzneimittelzulassung durchlaufen, wenn Daten, z. B. aus klinischen Studien, ihre Wirksamkeit belegen (sog. rationale Phytopharmaka). Beruht ihre Anwendung lediglich auf Anwendungserfahrungen, spricht man von traditionellen Phytopharmaka, die in Deutschland in einem als „Registrierung“ bezeichneten, vereinfachten Verfahren eine Vermarktungserlaubnis erlangen können [2]. Davon zu unterscheiden sind pflanzliche Präparate und NEM, die nicht dem Arzneimittelgesetz unterliegen.

In Deutschland ist der Vertrieb von pflanzlichen Präparaten ein attraktives Geschäftsfeld. Das Land ist in Europa einer der größten Märkte für diese Präparate, mit einen Umsatz von 1,4 Mrd. € im Jahr 2020, von denen 92 % für die Selbstmedikation ausgegeben wurden [3] Allerdings ist die Zahl der verkehrsfähigen und zugelassenen Phytopharmaka in den letzten 5 Jahren um über 20 % zurückgegangen. Im Jahr 2021 waren jedoch noch über 1300 phytotherapeutische Präparate im Handel [3]. Zunehmende gesetzliche Auflagen auf Bundes- und EU-Ebene führen dazu, dass sich Hersteller dafür entscheiden, pflanzliche Präparate nicht als Phytotherapeutika, sondern als NEM anzubieten [3].

Die Deutsche Gesellschaft für Rheumatologie und Klinische Immunologie (DGRh) hat im Jahr 2021 eine Kommission für Komplementäre Heilverfahren und Ernährung (KHE) ins Leben gerufen, welche Empfehlungen zur Anwendung komplementärer Heilverfahren bei Erkrankungen des rheumatischen Formenkreises erarbeitet. Nach Empfehlungen und Stellungnahmen zu ayurvedischer Medizin, Homöopathie und Ernährung [4] hat es sich die Kommission nun zur Aufgabe gemacht, die wissenschaftliche Evidenz zu Phytotherapeutika und pflanzlichen Präparaten für die Rheumatologie zu sichten und Empfehlungen für ihre Verwendung zu formulieren.

## Methodik

Mitglieder der oben genannten Kommission haben die wissenschaftliche Literatur zu ausgewählten frei verkäuflichen pflanzlichen Präparaten und Phytotherapeutika anhand von PubMed-Recherchen gesichtet und Möglichkeiten für ihre Anwendung in der Rheumatologie geprüft. Dazu wurde in einer Online-Sitzung am 08.02.2023 eine Liste von Substanzen erstellt, die in der Rheumatologie häufig Anwendung finden (meist als Selbstmedikation). Jedes Mitglied der Kommission führte daraufhin eine Literaturrecherche zu einem oder 2 Präparaten durch und fasste die Ergebnisse in einer vorgegebenen Matrix zusammen. Diese bearbeitete zu jedem Präparat die folgenden Punkte:

1. möglicher Wirkmechanismus, wirksame Substanzen, vorhandene Präparate,
2. Überblick über die wissenschaftliche Evidenz zur klinischen Wirksamkeit in der Literatur,
3. mögliche Anwendungen in der Rheumatologie inklusive zu erwartender positiver Effekte,
4. mögliche Nebenwirkungen und Limitationen,
5. abschließende Empfehlung der Kommission,
6. Literaturverzeichnis.

Recherchiert wurde sowohl zu pflanzlichen Präparaten und NEM (Borretschöl, *Rosa canina*, Rosmarin und Safran), traditionellen Phytopharmaka (Brennnesselpräparate, Zubereitungen von Weidenrinde und das Mischpräparat Phytodolor®) sowie zu Cannabispräparaten, von denen sowohl rationale als auch traditionelle Phytopharmaka sowie frei verkäufliche Zubereitungen existieren. Die Ergebnisse wurden im Umlaufverfahren überprüft und in 2 weiteren Online-Sitzungen der Kommission KHE konsentiert. Anschließend wurden die Ausarbeitungen dem Vorstand der Deutschen Gesellschaft für Rheumatologie (DGRh) übermittelt und von diesem kritisch gewürdigt. Nach letztmaliger Überarbeitung wurden die Empfehlungen am 01.10.2024 online zugänglich gemacht.

## Ergebnisse

Die ausführlichen Versionen der Literaturrecherchen sowie der daraus abgeleiteten Empfehlungen sind auf der Homepage der DGRh abrufbar: https://www.dgrh.de/Start/Publikationen/Empfehlungen/Komplement%C3%A4re-Methoden.html.

Aus Gründen der Übersichtlichkeit sind die Ausarbeitungen zu den Punkten 1 bis 4 hier nur in verkürzter Form dargestellt. Die abschließenden Empfehlungen der Kommission (Punkt 5) werden in voller Länge wiedergegeben. Eine Übersicht über die Empfehlungen in Kurzform liefert Tab. 1.

**Tab. 1:** Empfehlungen der KHE zur Phytotherapie

Präparat	Vermutete Wirksubstanzen	Anwendung bei		Sicherheitsaspekte	Anmerkungen
		Entzündlichen Gelenkerkrankungen	Arthrosen		
Borretschöl	Gamma-Linolensäure	Keine Empfehlung	Keine Empfehlung	Pflanze enthält Karzinogene	Öl anwendbar zur gesundheitsbewussten Ernährung
Brennnessel	Flavonoide, Karotinoide, Phytosterole	Keine Empfehlung	Keine *aktive *Empfehlung	Interaktionen mit VKA, Antidiabetika	–
Cannabis	Tetrahydrocannabinol (THC), Cannabidiol (CBD)	Keine Empfehlung	Keine Empfehlung	Abhängigkeit, Koordinationsstörungen	Evtl. bei neuropathischen Schmerzen und Schlafstörungen
Phytodolor®	Flavonoide, Salicin, Phenylglykoside	Keine Empfehlung	Keine *aktive *Empfehlung	Cave: Allergie, Schwangerschaft	Hoher Alkoholgehalt des Präparats
*Rosa canina*	Polyphenole, Flavonoide, Antioxidanzien	Keine Empfehlung	Keine *aktive *Empfehlung	Gel. Allergie, Toxizität gering	–
Rosmarin	Äther. Öle, Gerbstoffe, Flavonoide	Keine Empfehlung	Keine Empfehlung	Hohe Dosen leber-, neurotoxisch	–
Safran	Karotinoide, Bitterstoffe	Keine Empfehlung	Keine Empfehlung	Toxizität gering	–
Weidenrinde	Salicin, Gerbstoffe, Flavonoide	Keine Empfehlung	Keine *aktive *Empfehlung	Toxizität gering	Cave: Aspirin-Allergie

Keine *aktive* Empfehlung: Bei Wunsch der Patienten nach Selbstmedikation muss nicht abgeraten werden

### Behandlung rheumatischer Erkrankungen mit Borretsch sowie mit NEM oder Arzneimitteln auf der Basis von Borretschbestandteilen

Bor(r)etsch (*Borago officinalis*, auch Gurkenkraut, engl. „borage“) gehört zur Familie der Raublattgewächse (*Boraginaceae*). Als Heilpflanzen wurden v. a. Blüten in verschiedenen Indikationen verwendet (*Boraginis flos, Flores boraginis*), auch „Entzündungen“, „Rheumatismus“ und „Blutreinigung“ werden historisch benannt.

Aktuelle Studien zur Nutzung der gesamten Pflanze, Blätter oder Blüten wurden nicht gefunden. Die Pflanze enthält Pyrrolizidinalkaloide, die als gentoxisch, karzinogen und lebertoxisch eingestuft sind. Ihr Konsum sowie ihre pharmazeutische Anwendung sollten vermieden werden.

Borretschsamen bestehen zu einem Drittel aus Öl (Boraginis officinalis oleum raffinatum, Boraginis oleum, DAC). Dieses hat einen relevanten Anteil an Gamma-Linolensäure (GLA) sowie einer Reihe anderer Fettsäuren [5]. GLA ist eine Omega-6-Fettsäure, der antiinflammatorische Eigenschaften zugeschrieben werden [6]. Borretschsamenöl wird als NEM rezeptfrei über Apotheken vertrieben. Einige Präparate enthalten weitere Fettsäuren bzw. Vitamin E. Die Indikation lautet „zur Substitution ungesättigter Fettsäuren“.

#### Abschließende Empfehlung der Kommission

Die wissenschaftliche Evidenz reicht aus, um vor der regelmäßigen Anwendung von Borretschblättern und Blüten zu warnen. Die in Borretsch enthaltenen Pyrrolizidinalkaloide sind potenziell teratogen, kanzerogen und lebertoxisch. Die GLA als Bestandteil des Borretschsamenöls hat im Tierexperiment der kristallinduzierten Arthritis und der durch Freund-Adjuvans induzierten Arthritis bei Ratten und kleineren kontrollierten Studien bei Patienten mit rheumatoider Arthritis (RA) einen begrenzten antiphlogistischen Effekt und eine leichte Verbesserung des Lipidprofils gezeigt. Der Effekt setzt eine Tagesdosis von mindestens 1500 mg GLA voraus und ist vergleichbar dem Effekt von Fischöl. Eine Kombination der pflanzlichen und tierischen ungesättigten Fettsäuren ist nicht wirksamer als die Einzelsubstanz. Die Studien sind zwar teils systematisch, kontrolliert und mit validierten Outcomeparametern durchgeführt, aber nur mit sehr kleinen Patientenkollektiven, hohen Abbrecherraten und ohne Selektionsstrategie bei Abbrechern berechnet, eine Studie hat keinen Placeboarm. Aufgrund dieser mangelhaften Datenlage wird eine Anwendung in therapeutischer Intention nicht empfohlen.

Borretschsamenöl von zertifizierten Herstellern oder von anderen pflanzlichen Quellen gewonnene GLA kann als Alternative für andere Omega-3-Fettsäuren enthaltende Öle, Gemüse, Nüsse, Algen oder Fisch im Rahmen einer gesundheitsorientierten Ernährung entsprechend der Empfehlung der Kommission [4] eingesetzt werden, wenn Letztere z. B. bei Allergien nicht genutzt werden können.

Die publizierten Daten und der Zulassungsstatus rechtfertigen aus rheumatologischer Sicht keine additive Behandlung rheumatischer Erkrankungen mit Borretschsamenölpräparaten.

### Behandlung rheumatischer Erkrankungen mit Brennnesselpflanzen sowie mit NEM oder Arzneimitteln auf der Basis von Brennnesselbestandteilen

Brennnesseln (*Urtica*) bilden eine Pflanzengattung mit 30 bis 70 Unterarten. In Deutschland am meisten verbreitet sind die Große (*Urtica dioica*) und die Kleine Brennnessel (*Urtica urens*) sowie die Röhricht-Brennnessel (*Urtica kioviensis*).

Blätter, Stängel und Wurzeln werden in der Volksmedizin zur Wundbehandlung, als Diuretika, zur Behandlung von Gelenkschmerzen, als Anthelmintika und bei Atemwegserkrankungen benutzt (Übersicht in [7]). Mit der Indikation „Zur unterstützenden Behandlung rheumatischer Beschwerden“ werden in Deutschland mehrere Phytopharmaka mit Brennnesselblätter-Trockenextrakt sowie ein flüssiges Präparat als rezeptfreie, apothekenpflichtige Arzneimittel angeboten. Zusätzlich werden Brennnesselpräparate mit der Bezeichnung „Lebensmittel“ oder „NEM“ vertrieben.

Brennnesselblätter und -wurzeln enthalten in variablen Anteilen Flavonoide, Phenolsäuren, Carotinoide, organische Säuren und Phytosterole [7–9]. In-vitro-Experimente an verschiedenen Zellkulturen zeigten die Hemmung des proinflammatorischen Transkriptionsfaktors NF-kappaB (NF-κB) und weiterer Zytokine durch den Brennnesselblätter-Trockenextrakt IDS23 [10, 11].

Klinische Studien an Patienten mit Gelenkerkrankungen wurden sowohl mit frischen Brennnesselblättern als auch mit dem oben genannten Trockenextrakt durchgeführt. Zu Letzterem existieren lediglich Anwendungsbeobachtungen, die nicht in einem wissenschaftlichen Fachjournal publiziert worden sind.

Die Gefahr von Nebenwirkungen durch orale Brennnesselpräparate wird als nicht relevant eingeschätzt. Das allergene Potenzial von Brennnesselpollen ist sehr gering [12]. In den Fachinformationen wird die Anwendung in Schwangerschaft und Stillzeit sowie bei Kindern unter 12 Jahren nicht empfohlen, Brennnesselpresssaft nicht für Personen unter 18 Jahre. Die Fachinformationen zu Phytopharmaka auf der Basis von Brennnesseltrockenextrakt enthalten Warnhinweise zu Wechselwirkungen mit Antidiabetika und Vitamin-K-Antagonisten.

#### Abschließende Empfehlung der Kommission

Die wissenschaftliche Evidenz reicht nicht aus, um die Verschreibung und Anwendung von Brennnesselpräparaten für Patienten mit Symptomen aufgrund eines definierten entzündlich rheumatischen Krankheitsbildes zu empfehlen.

Die Effekte von Bestandteilen aus Brennnesselextrakten auf Entzündungsmediatoren in vitro erlauben keine Rückschlüsse darauf, dass entzündungshemmende Bestandteile der Pflanze in vivo wirksam werden. Systematische kontrollierte klinische Studien an Fallkollektiven mit klar umrissenen rheumatologischen Krankheitsbildern sind nicht vorhanden.

Besteht aus rheumatologischer Sicht keine Indikation für eine neue oder intensivierte antirheumatische Therapie, aber ein dringender Behandlungswunsch aufseiten des Patienten, muss von einer vom Patienten initiierten Behandlung mit den im Handel befindlichen Brennnesselpräparaten nicht abgeraten werden.

### *Cannabis sativa *und ihr Einsatz bei entzündlich rheumatischen Erkrankungen

Es gibt 3 Unterarten von Cannabis (Hanf): *Cannabis sativa, Cannabis indica* und *Cannabis ruderalis*. *Cannabis sativa* ist die am weitesten verbreitete Pflanze, die für kommerzielle und pharmazeutische Zwecke angebaut wird [13].

Identifiziert sind über 104 Phytocannabinoide als aktive Substanzen der Pflanze. Von pharmakologischem Interesse und gut untersucht sind die beiden Hauptinhaltsstoffe, das Delta9-Tetrahydrocannabinol (THC) und das Cannabidiol (CBD).

THC ist für die psychoaktiven Effekte von Cannabis verantwortlich, da es als partieller Agonist für Cannabinoidrezeptoren vom Typ 1 (CB1) wirkt [14]. CB1-Rezeptoren stellen die größte Gruppe von Bindungsstellen für Liganden im Zentralnervensystem dar, finden sich aber auch im Magen-Darm-Trakt, auf Makrophagen, Mastzellen und Keratinozyten [15, 16].

CBD weist eine sehr geringe Affinität für CB1- und CB2-Cannabinoidrezeptoren (CBR1 und CBR2) auf [17] und vermag CBR1 sowohl zu aktivieren als auch zu hemmen [18, 19]. CBD ist in der Lage, die Signalwirkung von Adenosinrezeptoren zu verstärken, was auf eine mögliche Rolle bei Schmerzen und Entzündungen schließen lässt [20]. Hanfsamenöl bietet sich zusätzlich aufgrund seines hohen Anteils an mehrfach ungesättigten Fettsäuren (PUFAs) und Vitamin E als Hautschutz an [21].

Als medizinisches Cannabis werden Zubereitungen aus den Inhaltsstoffen der Cannabispflanzen bezeichnet, welche medizinischen Anwendungen dienen. Cannabinoide sind eine heterogene Gruppe von Stoffen, die auf die Cannabinoidrezeptoren wirken. Dazu zählen auch synthetische Cannabinoide und Endocannabinoide. Bislang erfolgte die Anwendung von Cannabinoiden durch das „Gesetz zur Änderung betäubungsmittelrechtlicher und anderer Vorschriften“. Es umfasst die Verordnung von Cannabis in Form von getrockneten Blüten oder Extrakten in standardisierter Qualität und Arzneimittel mit den Wirkstoffen Dronabinol oder Nabilon (§ 31 Abs. 6 Satz 1 SGB V). Seit dem 01.04.2024 ist durch den Wegfall der Einordnung als Betäubungsmittel (BTM) eine neue Gesetzeslage entstanden. Lediglich Nabilon (reines synthetisches THC) ist nach wie vor BTM-pflichtig. An den Regeln zur Erstattungsfähigkeit von Cannabinoiden hat sich nichts geändert. Für die medizinische Anwendung sind zugelassen:

- Nabiximols (Sativex®, Allmiral Hermal GmbH, Deutschland): CBD 2,5 mg + THC 2,7 mg pro Sprühstoß, für die Therapie der mittelschweren bis schweren Spastik bei Erwachsenen mit multipler Sklerose,
- Cannabidiol (Epidyolex®, GW Pharma, Niederlande): 1 ml der Lösung = 100 mg Cannabidiol, für die adjuvante Behandlung von Krampfanfällen, im Zusammenhang mit dem Lennox-Gastaut-Syndrom (LGS) oder dem Dravet-Syndrom mit Clobazam bei Patienten ab 2 Jahren,
- Nabilon (Canemes®, AOP Orphan Pharmaceuticals, Deutschland): synthetisches THC, für die Behandlung von chemotherapiebedingter Emesis und Nausea bei Krebspatienten, die auf andere antiemetische Behandlungen nicht adäquat ansprechen,
- Dronabinol (Marinol®, Solvay Pharamceuticals, Deutschland, Syndros®, Benuvia Operations, Texas, U.S.A.): synthetisches THC, wird angewendet bei Kachexie/Anorexie und Appetitlosigkeit bei einer Krebserkrankung oder Aids (Zulassung in den USA und Kanada).

Frei verkäuflich sind Cannabiszubereitungen, z. B. CBD-Öl, in verschiedenen Konzentrationen (1–40 %) sowie Salben oder Gels für die topische Anwendung, die mit CBD angereichert sind.

Experimentelle Studien haben gezeigt, dass Cannabinoide analgetische und entzündungshemmende Wirkungen haben. Eine CBD-induzierte Unterdrückung der Zytokinproduktion wurde in In-vitro-Studien und Tiermodellen nachgewiesen [22–25]. Tierexperimentelle Studien mit kollageninduzierter Arthritis sprachen für eine immunsuppressive und antiarthritische Wirkung von CBD [26, 27].

Eine klinische Studie mit 58 RA-Patienten zeigte eine signifikante Verbesserung der Bewegungs- und Ruheschmerzen sowie des globalen Aktivitätsscores (DAS28) unter einer standardisierten Lösung von CBD/THC, wobei die Hauptwirkungen wahrscheinlich durch die THC-Komponente vermittelt wurden [28]. Eine 2021 publizierte Metaanalyse wertete 15 Beobachtungsstudien zu unterschiedlichen rheumatischen Erkrankungen aus [29]. Jeder sechste Patient hatte bereits Cannabis konsumiert. Cannabiskonsum war mit einer signifikanten Abnahme der VAS für Schmerz verbunden, bei Patienten mit Fibromyalgie (FMS) fanden sich Anzeichen für eine Verbesserung von Lebensqualität und depressionsbedingten Symptomen sowie der Schlafqualität. Eine Metaanalyse von Studien zur Anwendung von Cannabinoiden bei FMS stufte 5 Arbeiten (Beobachtungsstudien und Kohortenanalysen) als relevant ein, konstatierte jedoch trotz positiver Effekte schwerwiegende methodische Einschränkungen, sodass keine endgültige Empfehlung für diese Indikation ausgesprochen wurde [30]. Ein aktueller Review bestätigt diese skeptische Beurteilung [31]. Auch inhaliertes medizinisches Cannabis zeigte bei FMS nur eine geringe analgetische Potenz [32].

Die deutsche Cannabis-Begleiterhebung (04/2017–03/2022) wertete mehr als 10.000 Anwendungen aus [33]. Nebenwirkungen wie Müdigkeit und Schwindel waren häufig, aber in der Regel milde. Bei einem Drittel der Patienten wurde die Therapie vor Ablauf eines Jahres abgebrochen, hauptsächlich aufgrund fehlenden Effektes, bei 26 % der Fälle waren Nebenwirkungen der Abbruchgrund. Mit Cannabisblüten behandelte Patienten gaben seltener Nebenwirkungen an unter konventionellen Cannabis-Arzneimitteln und berichteten eine „euphorisierende Wirkung“ 3‑mal häufiger. Der Anteil an Diagnosen mit entzündlich rheumatischen Erkrankungen war mit 2,6 % klein (FMS *n* = 307, Spondylitis ankylosans *n* = 53, RA *n* = 82), sodass die Ergebnisse nicht für die Rheumatologie verallgemeinert werden können.

Klinische Studien mit hoch dosiertem CBD zur Behandlung von Epilepsie und psychiatrischen Erkrankungen berichten über CBD-induzierte Leberanomalien, Durchfall, Müdigkeit, Erbrechen und Schläfrigkeit [34]. Rifampicin, Carbamazepin oder Johanniskraut können die Plasmaspiegel von CBD senken. In der Fachinformation wird auf relevante Wechselwirkungen u. a. mit Warfarin, Omeprazol, Propofol und Lorazepam hingewiesen. Auch in der Fachinformation von Nabiximols wird auf Interaktionen z. B. mit Azolderivaten, Ritonavir, Clarithromycin hingewiesen. Darüber hinaus wurde eine längere Einnahme von Cannabinoiden mit der Entwicklung einer Abhängigkeit in Verbindung gebracht [35]. Bei prädisponierten Personen wurde ein erhöhtes Risiko für psychotische Störungen festgestellt [36].

#### Abschließende Empfehlung der Kommission

Für medizinischen Cannabis liegt keine ausreichende wissenschaftliche Evidenz aus klinischen Studien vor, die eine explizite Verordnung bei einer definierten entzündlich rheumatischen Erkrankung zur Krankheitsmodifikation oder zur symptomatischen Therapie begründet. Für den Einsatz bei (v. a. neuropathischen) Schmerzen, Schlafstörungen und zur Verbesserung der Lebensqualität liegen positive Daten aus der Behandlung chronischer Schmerzpatienten und neurologischer Erkrankungen vor. Diese können in Einzelfällen die Anwendung bei ausgewählten Patienten mit rheumatischen Erkrankungen im Rahmen eines therapeutischen Gesamtkonzepts rechtfertigen. Eine Verordnung kann dann im Rahmen der gesetzlichen Rahmenbedingungen der Verordnungsfähigkeit (§ 31 Abs. (6) Fünftes Buch Sozialgesetzbuch V) in Betracht gezogen werden.

Einschränkend ist zu erwähnen, dass Daten zu Dosis, Langzeiteffekten und Nebenwirkungen nicht in ausreichendem Maße vorliegen. Ein Abhängigkeitspotenzial sollte berücksichtigt werden. Bei chronischen Schmerzerkrankungen kann eine Opioidreduktion mit medizinischem Cannabis erzielt werden. Über das Nebenwirkungsprofil und Medikamenteninteraktionen sollen die verordnenden Ärzte und Patienten informiert sein.

### Einsatz von Phytodolor® bei rheumatischen Erkrankungen

Phytodolor® (Bayer Vital GmbH, Deutschland), (interne Abkürzung der Herstellerfirma: STW1) ist eine Tinktur aus Eschenrinde (*Fraxinus excelsior*), Zitterpappelrinde und -blättern (*Populus tremula*) und echtem Goldrutenkraut (*Solidago virgaurea*) in einem Mischungsverhältnis von 1:3:1. Als Hauptbestandteile der Eschenrinde sind Cumarin und Iridoid-Bitterstoffe angegeben, für die Zitterpappel Salicin/Salicortin und für die Goldrute Flavonoide, Triterpensaponine und Phenolglykoside.

Das Phytotherapeutikum ist laut Produktinformation als orale Anwendung bei Erwachsenen vorgesehen für „schmerzhafte Beschwerden bei degenerativen und entzündlichen rheumatischen Erkrankungen“. Die Dosierung wird mit 3‑mal 30 bis 40 Tropfen angegeben. Der Einsatz für mehr als 4 Wochen wird nicht empfohlen. Das Präparat wird rezeptfrei über die Apotheke vertrieben.

Für STW1 konnten in humanen Zellkulturen eine reduzierte TNF-alpha-Genexpression und Inhibierung der Synthese von COX‑2 sowie Veränderungen des Zytokinprofils nachgewiesen werden [37]. In tierexperimentellen Modellen wurden antiphlogistische, analgetische, antiexsudative und antiproliferative Effekte beschrieben [37–39].

Für die RA liegen lediglich Cochrane-Analysen der beiden vom Hersteller zur Verfügung gestellten unpublizierten Untersuchungen vor [40, 41]. Es handelt sich um 2 randomisierte Studien von 1987 über 2 Wochen und 1988 über 12 Monate. Hier zeigte sich zunächst ein höherer Diclofenac-Gebrauch unter Verum als unter Placebo. In den Monaten 2 bis 11 sank der Gebrauch von nichtsteroidalen Antirheumatika (NSAR) unter Therapie mit Phytodolor® unter den des Placeboarmes.

In einer weiteren Cochrane-Analyse zur Arthrose wurden 3 vom Hersteller durchgeführte Studien mit insgesamt 176 Probanden ausgewertet. Hier wurde das Präparat gegen Placebo oder Piroxicam getestet. Die Studien zeigten einen Rückgang des NSAR-Verbrauchs und als einzigen Outcomeparameter eine Besserung des Finger-Boden-Abstandes. Die Studien wurden wegen methodischer Schwächen als begrenzt aussagefähig („of limited use“) bezeichnet [42].

Eine Metaanalyse von 11 randomisierten, größtenteils unpublizierten Studien bei unterschiedlichen muskuloskeletalen Symptomen und Krankheitsbildern (Rückenschmerzen, Epikondylitis, RA, Spondylitis und Gonarthrose) zeigte keine signifikanten Effekte des Präparates. Über leichte unerwünschte Ereignisse wurde berichtet (8,1 % in der Placebogruppe, 14,2 % unter STW1 und 18,9 % bei NSAR-Einnahme) [43].

Der Beipackzettel des Präparates (über https://phytodolor.de) enthält Warnhinweise bezüglich der Anwendung in Schwangerschaft und Stillzeit, bei Magen- und Darmgeschwüren, Leber- und Nierenfunktionseinschränkungen. Der hohe Alkoholgehalt von 46,5 % wird dort ebenfalls angesprochen: „Ein gesundheitliches Risiko besteht u. a. bei Leberkranken, Alkoholkranken, Epileptikern, Patienten mit organischen Erkrankungen des Gehirns, Schwangeren, Stillenden und Kindern. Die Wirkung anderer Arzneimittel kann beeinträchtigt oder verstärkt werden.“ „Häufige Nebenwirkungen von Phytodolor® sind Magen-Darm-Beschwerden wie Magenschmerzen, Übelkeit und Erbrechen. Gelegentlich treten Kopfschmerzen auf. Es kann zu einem Anstieg des Blutzuckers, zu Überempfindlichkeitsreaktionen (Hautreaktionen), zu Wassereinlagerung (Ödem) und häufigem Wasserlassen kommen.“ (https://www.phytodolor.de/pflichttext)

#### Abschließende Empfehlung der Kommission

Es besteht keine ausreichende klinisch wissenschaftliche Evidenz zur Verordnung der Phytodolor®-Tinktur im Rahmen definierter entzündlich rheumatischer Erkrankungen. Die Studiendaten stammen aus den 80er- und 90er-Jahren mit variablen Studiendesigns und sind überwiegend nicht bzw. nur in Zusammenfassungen publiziert. Des Weiteren liegen keine Daten zur längerfristigen Einnahme und zum Einfluss auf den Langzeitverlauf rheumatischer Erkrankungen vor. Limitierend ist auch der nur über maximal 4 Wochen empfohlene Einsatz im Kontext chronischer Erkrankungen.

Bei Vorliegen leichter bis moderater Schmerzen bei degenerativen Gelenk- und Wirbelsäulenerkrankungen muss ein kurzfristiger Einsatz nicht zwingend ausgeschlossen werden, insbesondere dann, wenn NSAR oder andere pharmakologische Interventionen kontraindiziert oder aus anderen Gründen nicht angezeigt sind und wenn vonseiten der Patienten der Wunsch nach einer phytotherapeutischen Behandlung geäußert wird.

### Behandlung rheumatischer Erkrankungen mit *Rosa canina* (Hagebutte)

*Rosa canina*, auch als Hundsrose oder Wildrose bezeichnet, stammt aus der Familie der Rosengewächse (*Rosaceae*). Sie ist in verschiedenen Regionen Europas, Asiens und Nordafrikas beheimatet und wird in der traditionellen chinesischen Medizin verwendet. Einer Vielzahl von bioaktiven Verbindungen (Flavonoide, Polyphenole, Vitamin C, Carotinoide und ätherische Öle) [44–46] wurden die in vitro beobachteten antioxidativen, entzündungshemmenden und immunmodulatorischen Eigenschaften zugeschrieben [47]. Zu Letzteren gehören die Hemmung von COX‑1 und -2 [48] und die Inhibition von Interleukin-1α, Interleukin-1β und Tumornekrosefaktor [49].

Eine klinische Studie zur RA umfasste 20 RA-Patientinnen und 10 gesunde Probanden, die über 28 Tage 10,5 g *Rosa canina* 1‑mal täglich als Pulver erhielten. Die Studie zeigte weder eine signifikante Änderung des C‑reaktiven Proteins (CRP) noch Veränderungen in den erfassten antioxidativen Enzymen [50]. Klinische Parameter wurden nicht bestimmt.

In einer weiteren doppelblinden, placebokontrollierten Studie an 89 RA-Patienten wurde mit 5 g Hagebuttenpulver über 6 Monate behandelt [51]. Veränderungen im DAS28 waren zwischen Placebo und Verum nicht signifikant verschieden, eine minimale Verbesserung des Gelenkfunktionsindex HAQ um 3–4 % erscheint nicht relevant.

Zur Behandlung von Arthrosen mit *Rosa canina* identifizierte eine PubMed-Recherche 6 klinische Studien. In einer Studie, an der Mitarbeiter der Herstellerfirma maßgeblich beteiligt waren, wurden 92 Patienten mit einer Gonarthrose mit einem Mischpräparat aus *Rosa canina*, Brennnessel und Teufelskralle oder mit Placebo behandelt [52]. Die Wirksamkeit wurde anhand des WOMAC-Schmerz-Scores (Western Ontario and McMaster Universities Arthritis Index) bewertet. In der Verumgruppe fiel die Reduktion des WOMAC-Schmerz-Scores deutlich höher aus als in der Placebogruppe.

Die Wirkung von *Rosa canina* in Reinform auf Gon- und Koxarthrose wurde in einer placebokontrollierten, doppelblinden Crossover-Studie über 3 Monate untersucht [53]. Die Verumgruppe zeigte nach 3 Wochen unter 1‑mal täglicher Gabe von 5 g *Rosa canina* eine signifikante Reduktion des WOMAC-Schmerz-Scores im Vergleich zur Placebogruppe. Nach 3 Monaten war der Unterschied nicht mehr signifikant. In der Verumgruppe reduzierte sich die Einnahme von Paracetamol, nicht jedoch von NSAR [53]. In einer Studie zur radiologisch gesicherten Gon- oder Koxarthrose zeigten sich eine Verbesserung der Mobilität des Hüftgelenks und ein signifikanter Rückgang der Schmerzen unter 2‑mal 2,5 g *Rosa-canina*-Pulver im Vergleich zu Placebo sowie ein Rückgang der NSAR-Einnahme [54]. Auch eine Studie an 112 Betroffenen mit unterschiedlichen Arthrosen oder degenerativen Veränderungen der Halswirbelsäule demonstrierte eine Linderung des Arthroseschmerzes im Vergleich zu Placebo [55]. An allen Arthrosestudien war die Herstellerfirma beteiligt. Eine Metaanalyse zur Wirksamkeit von *Rosa canina* für die Arthrose attestierte den oben genannten Studien eine gering bis mäßig starke, kurzzeitige Reduktion des Arthroseschmerzes, wobei darauf hingewiesen wurde, dass Wirksamkeit und Sicherheit an großen Kohorten evaluiert werden sollten [56]. Weder in der Leitlinie des American College of Rheumatology zur Behandlung der Hand‑, Hüft- und Kniearthrose noch in den deutschen S2k-Leitlinien zur Kox- und Gonarthrose werden Empfehlungen zur Behandlung mit *Rosa canina* ausgesprochen.

Studiendaten zu Vaskulitiden und Kollagenosen liegen nicht vor.

Als mögliche Nebenwirkungen einer Therapie mit *Rosa canina* werden Kopfschmerzen, Magen-Darm-Störungen, Schluckbeschwerden, Erkältungen, Harnwegsinfektionen, allergische Vaskulitis, Hautausschlag bzw. Ekzem und Gewichtszunahme beschrieben.

#### Abschließende Empfehlung der Kommission

*Rosa canina* zeigt keinen signifikanten Einfluss auf die Krankheitsaktivität einer rheumatoiden Arthritis und ist somit für die Behandlung dieser Erkrankung nicht zu empfehlen.

Bei Arthrosen kann durch den Einsatz von *Rosa canina* eine gering bis mäßig starke, kurzzeitige Linderung des Schmerzes erzielt werden. Der Mangel an herstellerunabhängigen Studien und die geringe Stichprobenzahl sowie die fehlende Abbildung in Leitlinien stehen einer Empfehlung der Kommission zum jetzigen Zeitpunkt im Wege. Bei einem Wunsch nach Selbstmedikation muss von einer Anwendung bei degenerativen Gelenkerkrankungen nicht abgeraten werden.

### Behandlung rheumatischer Erkrankungen mit Rosmarin sowie mit NEM oder Arzneimitteln auf der Basis von Rosmarinbestandteilen

Der Rosmarin (*Salvia rosmarinus*; Synonym *Rosmarinus officinalis*) ist eine Art der Gattung *Salvia* und ein immergrüner Halbstrauch aus der Familie der Lippenblütler (*Lamiaceae*).

In Deutschland sind etwa 300 Präparate mit Rosmarin erhältlich. Als pflanzliche rezeptfreie, apothekenpflichtige Präparate werden mehrere Rosmarinprodukte als Salbe, Tropfen oder ätherisches Öl ohne diagnosebezogene Indikationen angeboten.

Rosmarin enthält zwischen 1 und 2,5 % ätherische Öle zusammen mit verschiedenen Terpenverbindungen (z. B. Carnosol und Rosmanol), des Weiteren Gerbstoffe (hauptsächlich Rosmarinsäure), Flavonoide, Glykolsäure, Kaffeesäure, Bitterstoffe und Saponine.

Eine signifikante antiinflammatorische Wirkung durch die Hemmung der Neutrophilenaktivität und die Modulation der NF-κB-Aktivität wurde durch Carnosolsäure (CA) und Rosmarinsäure im Tiermodell beschrieben [57]. CA, Carnosol und Rosmanol verhindern im Modell die Expression von proinflammatorischen Zytokinen, Adhäsionsmolekülen, Chemokinen und Prostaglandinen [58, 59]. CA hemmt die Osteoklastogenese und Knochenresorption sowie die Aktivierung von Proteinkinasen in vitro und im Tiermodell [60], Rosmanol und Carnosol haben im Tiermodell antiarthritische Eigenschaften [61].

Zu rheumatologischen Erkrankungen oder Gelenkschmerzen sind nur wenige klinische Studien vorhanden. Die Mehrzahl verwendet Rosmarin in Mischpräparaten mit anderen Phytopräparaten wie Salbei, Melisse oder *Rosa canina* [62].

In einer 8‑wöchigen Anwendungsbeobachtung (AWB) wurde die Wirksamkeit eines aus Hopfen, Rosmarinextrakt und Oleanolsäure hergestellten Präparats bei Patienten mit Arthrose, RA und FMS untersucht. Erfasst wurden Schmerzen mithilfe einer visuellen Analogskala (VAS), eine verkürzte Skala zur Messung der Arthritis (AIMS2) und ein FMS-Fragebogen. Bei Patienten mit Arthrose und RA wurde ein statistisch signifikanter Rückgang der Schmerzen um 50 % bzw. 40 % ohne schwerwiegende Nebenwirkungen beobachtet [63]. Dennoch sollten die Ergebnisse kritisch gesehen werden (nicht kontrolliertes Studiendesign, durch den Hersteller des Phytopräparates gesponsert, keine weiteren Studien des Präparates bei RA bzw. Arthrose seit 2005).

#### Abschließende Empfehlung der Kommission

Die Effekte von Bestandteilen aus Rosmarin auf Entzündungsmediatoren in vitro und in vivo schließen ein mögliches therapeutisches Potenzial nicht aus. Systematische kontrollierte klinische Studien von Rosmarinpräparaten an Fallkollektiven mit klar definierten rheumatologischen Krankheitsbildern sind jedoch nicht vorhanden. Die wissenschaftliche Evidenz reicht daher nicht aus, um die Verschreibung und Anwendung von Rosmarinpräparaten für Patienten mit entzündlich rheumatischen Krankheitsbildern zu empfehlen.

### Behandlung rheumatischer Erkrankungen mit Safran

Der Safran (von arabisch/persisch „das Gelbe“, „Safran“), *Crocus sativus*, ist eine Krokusart, die v. a. als Gewürzpflanze genutzt wird. Aus den Narben ihrer Blüten wird das ebenfalls Safran genannte Gewürz gewonnen. Angebaut wird Safran unter anderem in Afghanistan, Iran, Marokko und der Türkei sowie in Österreich.

In Deutschland sind 46 Präparate mit Safran erhältlich. Als pflanzliche rezeptfreie, apothekenpflichtige Präparate werden sie z. B. in Kapselform ohne diagnosespezifische Indikation verkauft.

Safran enthält Karotinoide (Crocin, Crocetin), sodass sich mit Safran gewürzte Gerichte intensiv goldgelb färben. Weiter enthält er den Bitterstoff Safranbitter, aus dem sich beim Trocknen das für das Safranaroma verantwortliche Aldehyd Safranal bildet. In einem Tiermodell der kollageninduzierten Arthritis (CIA) führte die Behandlung mit Crocin zur Senkung der Plasmaspiegel von TNF‑α, IL-1β und IL‑6 [64]. In einem weiteren Tiermodell verbesserte Crocin die Knorpelregeneration und hemmte die Expression der Matrixmetalloproteinasen (MMP)-1-, -3- und -13-Gene [65].

Eine Metaanalyse wertete alle klinischen Studien zu *Crocus sativus* bei rheumatischen Erkrankungen bis Oktober 2021 aus [66], darunter 2 Studien zur RA [67, 68] und eine zum FMS [69]. Die Autoren attestierten den meisten Studien ein fehlerhaftes Design und konstatierten, dass die Evidenz weder für den Nachweis einer Wirksamkeit noch einer Unwirksamkeit ausreicht. Die Gefahr von relevanten Nebenwirkungen durch orale Safranpräparate wird als gering eingeschätzt.

#### Abschließende Empfehlung der Kommission

Die wissenschaftliche Evidenz reicht nicht aus, um die Verschreibung und Anwendung von Safranpräparaten für Patienten mit entzündlich rheumatischen Erkrankungen zu empfehlen.

### Behandlung rheumatischer Erkrankungen mit Weidenrinde sowie mit NEM oder Arzneimitteln auf der Basis von Weidenrindenbestandteilen

Weiden sind Bäume und Sträucher mit meist länglichen bis lanzettartigen Blättern, die zur Familie der Weidengewächse mit ca. 500 Arten gehören. Ihre wirksamen Inhaltstoffe werden meist aus der Silberweide (*Salix alba*), der Purpurweide (*Salix purpurea*) oder Reifweide (*Salix daphnoides*) gewonnen. Die Weidenrinde enthält neben Salizylsäure Derivate des Salicins (z. B. Salicortin und Tremulacin), Flavonoide, Gerbstoffe sowie Kaffeesäurederivate.

In der traditionellen Heilkunde werden Weidenrindenextrakte als Tee, Tinktur, Trockenextrakt oder Aufguss eingesetzt, aber auch in Kapseln als NEM vertrieben. Auch Kombinationspräparate mit Teufelskralle und/oder Brennnessel sind erhältlich. In der Naturheilkunde werden Weidenrindenextrakte heute meist für folgende so bezeichnete Indikationen eingesetzt: rheumatische Beschwerden, Gicht, Rückenschmerzen, Kopfschmerzen, Menstruationsbeschwerden.

Sowohl in vitro als auch in Tiermodellen hemmten Weidenrindenextrakte COX‑1, COX‑2, LOX, Zytokine (Interleukin-1β, TNF-α), Elastase, Hyaluronidase und zeigten eine antioxidative Wirkung. An diesen Effekten scheinen neben Salicylaten auch Polyphenole, Flavonoide und Gerbstoffe beteiligt zu sein [70–73].

Bei RA-Patienten konnte eine kleine, placebokontrollierte Studie zu 240 mg Weidenextrakt nach 6‑wöchiger Therapie keinen signifikanten Effekt nachweisen [41]. Weitere Studien zur RA oder anderen entzündlich rheumatischen Erkrankungen liegen nicht vor.

Bei 78 Arthrosepatienten wurde der schmerzlindernde Effekt von 240 mg Salicin/Tag über 2 Wochen im Vergleich zu Placebo untersucht. Der primäre Endpunkt war die Schmerzstärke (WOMAC-Osteoarthritis-Index). In diesem Parameter sowie in der Gesamtbewertung durch Arzt und Patient zeigte sich gegenüber dem Placebo eine mäßig schmerzlindernde Wirkung bei guter Verträglichkeit [74]. In einer weiteren Studie über 6 Wochen konnte von der gleichen Arbeitsgruppe bei 127 Arthrosepatienten jedoch kein statistisch signifikanter Unterschied im WOMAC-Schmerz-Score zwischen Weidenrindenextrakt und Placebo gefunden werden [75]. In einer vom Hersteller geförderten AWB von 90 Patienten mit Gon- oder Koxarthrose, die entweder ein standardisiertes Weidenrindenextraktpräparat oder eine konventionelle analgetische Therapie erhielten, beurteilten Ärzte und Patienten die Wirksamkeit in beiden Gruppen nach 6 Wochen als vergleichbar, der Wirkungseintritt bei den Weidenrindenextrakten war im Vergleich zur Standardtherapie verzögert [76].

Bei chronischen Rückenschmerzen schlussfolgert eine Cochrane-Analyse, dass tägliche Dosen von *Salix alba* standardisiert auf 120 mg oder 240 mg Salicin wahrscheinlich besser als Placebo eine kurzfristige Verbesserung der Schmerzen bewirken bzw. relativ gleichwertig zu 12,5 mg Rofecoxib sein könnten. Die Evidenz wurde aber als sehr schwach eingestuft [77].

In einer AWB zur Langzeitverträglichkeit von Weidenrindenextrakt an 436 Patienten mit chronischen Rückenschmerzen und Arthrose fand sich eine gute Verträglichkeit innerhalb von 34 Wochen. Relevante Wechselwirkungen mit NSAR oder Opioiden wurden nicht beobachtet [78]. Eine Risikobewertung von Silberweide (*Salix alba*) in Lebensmitteln des Deutschen Bundesinstituts für Risikobewertung stellt fest, dass nur sehr wenige Daten zur Toxizität verfügbar sind. Anaphylaktische Reaktionen können bei einer Allergie gegen Salicylate auftreten. Andere unerwünschte Wirkungen werden als wenig relevant angesehen [79].

Im US-amerikanischen Pharmacopeia Safety Review (USP) wird konstatiert: Nicht für Kinder, schwangere oder stillende Frauen oder Personen mit bekannter Aspirin-Überempfindlichkeit geeignet [80].

#### Abschließende Empfehlung der Kommission

Die wissenschaftliche Evidenz reicht nicht aus, um die Verschreibung und Anwendung von Weidenrindenextrakten für Patienten mit Symptomen aufgrund eines definierten entzündlich rheumatischen Krankheitsbildes zu empfehlen. Die Effekte von Bestandteilen aus Weidenrindenextrakten auf Entzündungsmediatoren in vitro erlauben keine Rückschlüsse darauf, dass entzündungshemmende Bestandteile der Pflanze in vivo wirksam werden. Größere systematische kontrollierte klinische Studien an Fallkollektiven mit klar umrissenen rheumatologischen Krankheitsbildern sind nicht vorhanden.

Eine leichte kurzfristige analgetische Wirkung wurde bei Arthroseschmerzen berichtet. Besteht daher aus rheumatologischer Sicht keine Indikation für eine neue oder intensivierte antirheumatische oder analgetische Therapie, aber ein dringender Behandlungswunsch aufseiten des Patienten, muss von einer vom Patienten initiierten Behandlung mit den im Handel befindlichen Weidenrindenpräparaten nicht abgeraten werden.

## Diskussion

Die hier von der Kommission für Komplementäre Heilverfahren und Ernährung erarbeiteten Empfehlungen stellen lediglich eine Momentaufnahme dar. Die Kommission erhebt keinen Anspruch darauf, sämtliche in der Rheumatologie verwendeten Phytotherapeutika und pflanzlichen Präparate berücksichtigt zu haben. So fehlen Daten zur Teufelskralle (*Harpagophytum*), zur Dreiflügelfrucht (*Tripterygium wilfordii*) und zum Bergwohlverleih (*Arnica montana*). Diese und weitere Heilpflanzen werden Gegenstand künftiger Recherchen der Kommission sein. Anwendungen von Weihrauch (*Boswellia*) und Gelbwurz (*Curcuma*) haben in die hier vorgestellte Liste von pflanzlichen Präparaten und Phytotherapeutika ebenfalls keinen Eingang gefunden. Beide Naturstoffe wurden von der Kommission bereits bei den Empfehlungen zur ayurvedischen Medizin ausführlicher erwähnt [4]. Ebenso wenig berücksichtigt wurden Heilpflanzen, die in anderen Ländern etabliert, aber in Deutschland nicht als Phytopharmaka oder pflanzliche Präparate verfügbar sind. So werden nach einem Bericht einer gemeinsamen Expertenkommission des Bundesamts für Verbraucherschutz und Lebensmittelsicherheit und des BfArM vom Januar 2020 allein in Indien ca. 7000 Pflanzen zur Therapie verschiedenster Erkrankungen genutzt und 700 in ayurvedischen Medizinsystemen gelistet (https://www.bfarm.de/SharedDocs/Downloads/DE/Arzneimittel/Zulassung/ZulRelThemen/abgrenzung/Expertenkommission/stellungnahmen/2020-01.pdf?__blob=publicationFile). Die Mehrzahl davon ist in der westlichen Kultur unbekannt oder wird sogar als Giftpflanze eingestuft (z. B. die Brechnuss, *Strychnos nux vomica*, oder Eisenhutgewächse, *Aconitum spp.*).

Auch bei den in Deutschland verfügbaren Präparaten ist nicht jede Anwendung als risikofrei anzusehen. Bei etablierten Phytopharmaka gibt die Packungsbeilage Auskunft über potenzielle Nebenwirkungen und Interaktionen. Aber auch die Einstufung eines pflanzlichen Präparates als NEM bedeutet nicht in jedem Fall, dass unerwünschte Ereignisse bei den Konsumenten ausgeschlossen sind [81].

Zusammenfassend kann konstatiert werden, dass der Stellenwert der hier vorgestellten pflanzlichen Präparate für die entzündlich rheumatischen und systemisch autoimmunen Erkrankungen vernachlässigbar ist. Im Hinblick auf degenerative Erkrankungen und auf die Therapie rheumatischer Symptome müssen die hier besprochenen Substanzen allerdings differenzierter betrachtet werden. Auch hier ist die Studienlage in aller Regel schlecht, aber es gilt das Prinzip: „Absence of evidence is not evidence of absence.“ Die Kommission hat bei den degenerativen Gelenkerkrankungen versucht, diesem Fakt bei einigen Präparaten durch eine Abstufung der Empfehlung Rechnung zu tragen. Es ist vorstellbar, dass zukünftige Studien zuverlässigere Aussagen zum Stellenwert der pflanzenbasierten Medizin erlauben. Dem steht jedoch der gewachsene bürokratische, organisatorische und finanzielle Aufwand bei der Planung und Durchführung klinischer Studien entgegen, der die Erfolgsaussichten nichtkommerzieller Initiativen erschwert [82] und auch für die meisten Hersteller pflanzlicher Präparate und Phytopharmaka schwer zu bewältigen sein dürfte. Daher wären firmenunabhängige Studien als „investigator initiated trials“ (IIT, Prüfer-initiierte Studien) sehr zu begrüßen, um die wissenschaftliche Datenlage zu verbessern.

## References

[1] Paracelsus PP (2006) Ann Pharm Fr 0(6):75294–75294. 10.1016/s0003-4509

[2] Beer AM, Schilcher H, Loew D (2013) Herbal medicines alternative to synthetical medicines. MMW Fortschr Med 155(Suppl 4):97–99 24934061

[3] Armbrüster NR‑E (2021) R. Fit für die Zukunft! Phytopharm Müssen Attraktiver Werden Z Phytother 42:95–99. 10.1055/a-1336-6262

[4] Keysser G, Michalsen A, Reuss-Borst M et al (2023) Recommendations of the committee on complementary medicine and nutrition in ayurvedic medicine, homeopathy, nutrition and Mediterranean diet. Z Rheumatol 82:517–531. 10.1007/s00393-023-01356-z 37212842 10.1007/s00393-023-01356-z PMC10382356

[5] Zurier RB, Rossetti RG (1996) gamma-Linolenic acid treatment of rheumatoid arthritis. A randomized, placebo-controlled trial. Arthritis Rheum 39:1808–1817. 10.1002/art.1780391106 8912502 10.1002/art.1780391106

[6] Kapoor R, Huang YS (2006) Gamma linolenic acid: an antiinflammatory omega‑6 fatty acid. Curr Pharm Biotechnol 7:531–534. 10.2174/138920106779116874 17168669 10.2174/138920106779116874

[7] Grauso L, de Falco B, Lanzotti V, Motti R (2020) Stinging nettle, Urtica dioica L.: botanical, phytochemical and pharmacological overview. Phytochem Rev 19:1341–1377. 10.1007/s11101-020-09680-x

[8] Devkota HP, Paudel KR, Khanal S et al (2022) Stinging Nettle (Urtica dioica L.): Nutritional Composition, Bioactive Compounds, and Food Functional Properties. Molecules. 10.3390/molecules27165219 36014458 10.3390/molecules27165219 PMC9413031

[9] Otles S, Yalcin B (2012) Phenolic compounds analysis of root, stalk, and leaves of nettle. ScientificWorldJournal 2012:564367. 10.1100/2012/564367 22593694 10.1100/2012/564367 PMC3349212

[10] Riehemann K, Behnke B, Schulze-Osthoff K (1999) Febs Lett 9(8):1622–1626. 10.1016/s0014-5793 10.1016/s0014-5793(98)01622-6 9923611

[11] Klingelhoefer S, Obertreis B, Quast S, Behnke B (1999) Antirheumatic effect of IDS 23, a stinging nettle leaf extract, on in vitro expression of T helper cytokines. J Rheumatol 26:2517–2522 10606356

[12] Vega-Maray AM, Fernandez-Gonzalez D, Valencia-Barrera R, Suarez-Cervera M (2006) Allergenic proteins in Urtica dioica, a member of the Urticaceae allergenic family. Ann Allergy Asthma Immunol 1(0):60799–60795. 10.1016/S1081-1206 10.1016/S1081-1206(10)60799-5 17042140

[13] Andre CM, Hausman JF, Guerriero G (2016) Cannabis sativa: the plant of the thousand and one molecules. Front Plant Sci 7:19. 10.3389/fpls.2016.00019 26870049 10.3389/fpls.2016.00019 PMC4740396

[14] Bloomfield MAP, Hindocha C, Green SF et al (2019) The neuropsychopharmacology of cannabis: A review of human imaging studies. Pharmacol Ther 195:132–161. 10.1016/j.pharmthera.2018.10.006 30347211 10.1016/j.pharmthera.2018.10.006 PMC6416743

[15] Pertwee RG (2008) The diverse CB1 and CB2 receptor pharmacology of three plant cannabinoids: delta9-tetrahydrocannabinol, cannabidiol and delta9-tetrahydrocannabivarin. British J Pharmacology 153:199–215. 10.1038/sj.bjp.0707442 10.1038/sj.bjp.0707442 PMC2219532 17828291

[16] Mackie K (2006) Cannabinoid receptors as therapeutic targets. Annu Rev Pharmacol Toxicol 46:101–122. 10.1146/annurev.pharmtox.46.120604.141254 16402900 10.1146/annurev.pharmtox.46.120604.141254

[17] Thomas A, Baillie GL, Phillips AM et al (2007) Cannabidiol displays unexpectedly high potency as an antagonist of CB1 and CB2 receptor agonists in vitro. British J Pharmacology 150:613–623. 10.1038/sj.bjp.0707133 10.1038/sj.bjp.0707133 PMC2189767 17245363

[18] Laprairie RB, Bagher AM, Kelly ME, Denovan-Wright EM (2015) Cannabidiol is a negative allosteric modulator of the cannabinoid CB1 receptor. British J Pharmacology 172:4790–4805. 10.1111/bph.13250 10.1111/bph.13250 PMC4621983 26218440

[19] De Petrocellis L, Orlando P, Moriello AS et al (2012) Cannabinoid actions at TRPV channels: effects on TRPV3 and TRPV4 and their potential relevance to gastrointestinal inflammation. Acta Physiol (oxf) 204:255–266. 10.1111/j.1748-1716.2011.02338.x 21726418 10.1111/j.1748-1716.2011.02338.x

[20] Carrier EJ, Auchampach JA, Hillard CJ (2006) Inhibition of an equilibrative nucleoside transporter by cannabidiol: a mechanism of cannabinoid immunosuppression. Proc Natl Acad Sci U S A 103:7895–7900. 10.1073/pnas.0511232103 16672367 10.1073/pnas.0511232103 PMC1472541

[21] Vaughn AR, Clark AK, Sivamani RK, Shi VY (2018) Natural Oils for Skin-Barrier Repair: Ancient Compounds Now Backed by Modern Science. Am J Clin Dermatol 19:103–117. 10.1007/s40257-017-0301-1 28707186 10.1007/s40257-017-0301-1

[22] Hobbs JM, Vazquez AR, Remijan ND et al (2020) Evaluation of pharmacokinetics and acute anti-inflammatory potential of two oral cannabidiol preparations in healthy adults. Phytother Res 34:1696–1703. 10.1002/ptr.6651 32147925 10.1002/ptr.6651

[23] Kaplan BL, Springs AE, Kaminski NE (2008) The profile of immune modulation by cannabidiol (CBD) involves deregulation of nuclear factor of activated T cells (NFAT). Biochem Pharmacol 76:726–737. 10.1016/j.bcp.2008.06.022 18656454 10.1016/j.bcp.2008.06.022 PMC2748879

[24] Nichols JM, Kaplan BLF (2020) Immune responses regulated by Cannabidiol. Cannabis Cannabinoid Res 5:12–31. 10.1089/can.2018.0073 32322673 10.1089/can.2018.0073 PMC7173676

[25] Kozela E, Juknat A, Kaushansky N et al (2013) Cannabinoids decrease the th17 inflammatory autoimmune phenotype. J Neuroimmune Pharmacol 8:1265–1276. 10.1007/s11481-013-9493-1 23892791 10.1007/s11481-013-9493-1

[26] Sumariwalla PF, Gallily R, Tchilibon S et al (2004) A novel synthetic, nonpsychoactive cannabinoid acid (HU-320) with antiinflammatory properties in murine collagen-induced arthritis. Arthritis Rheum 50:985–998. 10.1002/art.20050 15022343 10.1002/art.20050

[27] Malfait AM, Gallily R, Sumariwalla PF et al (2000) The nonpsychoactive cannabis constituent cannabidiol is an oral anti-arthritic therapeutic in murine collagen-induced arthritis. Proc Natl Acad Sci U S A 97:9561–9566. 10.1073/pnas.160105897 10920191 10.1073/pnas.160105897 PMC16904

[28] Blake DR, Robson P, Ho M et al (2006) Preliminary assessment of the efficacy, tolerability and safety of a cannabis-based medicine (Sativex) in the treatment of pain caused by rheumatoid arthritis. Rheumatology 45:50–52. 10.1093/rheumatology/kei183 16282192 10.1093/rheumatology/kei183

[29] Guillouard M, Authier N, Pereira B et al (2021) Cannabis use assessment and its impact on pain in rheumatologic diseases: a systematic review and meta-analysis. Rheumatology 60:549–556. 10.1093/rheumatology/keaa534 33159797 10.1093/rheumatology/keaa534

[30] Cameron EC, Hemingway SL (2020) Cannabinoids for fibromyalgia pain: a critical review of recent studies (2015–2019). J Cannabis Res 2:19. 10.1186/s42238-020-00024-2 33526114 10.1186/s42238-020-00024-2 PMC7819299

[31] Strand NH, Maloney J, Kraus M et al (2023) Cannabis for the treatment of fibromyalgia: a systematic review. Biomedicines. 10.3390/biomedicines11061621 37371716 10.3390/biomedicines11061621 PMC10295750

[32] van de Donk T, Niesters M, Kowal MA et al (2019) An experimental randomized study on the analgesic effects of pharmaceutical-grade cannabis in chronic pain patients with fibromyalgia. Pain 160:860–869. 10.1097/j.pain.0000000000001464 30585986 10.1097/j.pain.0000000000001464 PMC6430597

[33] Schmidt-Wolf G, Cremer-Schaeffer P (2021) Three years of cannabis as medicine-preliminary results of the survey accompanying the prescription of medical cannabis in Germany. Bundesgesundheitsblatt Gesundheitsforschung Gesundheitsschutz 64:368–377. 10.1007/s00103-021-03285-1 33564897 10.1007/s00103-021-03285-1 PMC7932947

[34] Huestis MA, Solimini R, Pichini S et al (2019) Cannabidiol Adverse Effects and Toxicity. Curr Neuropharmacol 17:974–989. 10.2174/1570159X17666190603171901 31161980 10.2174/1570159X17666190603171901 PMC7052834

[35] Schlag AK, Hindocha C, Zafar R et al (2021) Cannabis based medicines and cannabis dependence: A critical review of issues and evidence. J Psychopharmacol 35:773–785. 10.1177/0269881120986393 33593117 10.1177/0269881120986393 PMC8278552

[36] Hall W (2009) The adverse health effects of cannabis use: what are they, and what are their implications for policy? Int J Drug Policy 20:458–466. 10.1016/j.drugpo.2009.02.013 19362460 10.1016/j.drugpo.2009.02.013

[37] Ulrich-Merzenich G, Hartbrod F, Kelber O et al (2017) Salicylate-based phytopharmaceuticals induce adaptive cytokine and chemokine network responses in human fibroblast cultures. Phytomedicine 34:202–211. 10.1016/j.phymed.2017.08.002 28899503 10.1016/j.phymed.2017.08.002

[38] Gundermann KJ, Muller J, Kraft K (2020) STW1 and Its Versatile Pharmacological and Clinical Effects in Rheumatic Disorders: A Comprehensive Report. Evid Based Complement Alternat Med 2020:7841748. 10.1155/2020/7841748 32733586 10.1155/2020/7841748 PMC7376409

[39] el-Ghazaly M, Khayyal MT, Okpanyi SN, Arens-Corell M (1992) Study of the anti-inflammatory activity of Populus tremula, Solidago virgaurea and Fraxinus excelsior. Arzneimittelforschung 42:333–336 1497695

[40] Cameron M, Gagnier JJ, Chrubasik S (2011) Herbal therapy for treating rheumatoid arthritis. Cochrane Database Syst Rev 1002(14651858):CD2948–pub2. 10.1002/14651858.CD002948.pub2:10.1002/14651858.CD002948.pub2 PMC13120739 21328257

[41] Cameron M, Gagnier JJ, Little CV et al (2009) Evidence of effectiveness of herbal medicinal products in the treatment of arthritis. Part 2: Rheumatoid arthritis. Phytother Res 23:1647–1662. 10.1002/ptr.3006 19941324 10.1002/ptr.3006

[42] Cameron M, Gagnier JJ, Little CV et al (2009) Evidence of effectiveness of herbal medicinal products in the treatment of arthritis. Phytother Res 23:1497–1515. 10.1002/ptr.3007 19856319 10.1002/ptr.3007

[43] Uehleke B, Brignoli R, Rostock M et al (2011) Phytodolor(R) in musculoskeletal disorders: re-analysis and meta-analysis. Forsch Komplementmed 18:249–256. 10.1159/000332820 22105037 10.1159/000332820

[44] Pekacar S, Bulut S, Özüpek B, Orhan DD (2021) Anti-Inflammatory and Analgesic Effects of Rosehip in Inflammatory Musculoskeletal Disorders and Its Active Molecules. Curr Mol Pharmacol 14:731–745. 10.2174/1874467214666210804154604 34348637 10.2174/1874467214666210804154604

[45] Gruenwald J, Uebelhack R, Moré MI (2019) Rosa canina—Rose hip pharmacological ingredients and molecular mechanics counteracting osteoarthritis—A systematic review. Phytomedicine 60:152958. 10.1016/j.phymed.2019.152958 31138475 10.1016/j.phymed.2019.152958

[46] Rossnagel K, Willich SN (2001) Value of complementary medicine exemplified by rose-hips. Gesundheitswesen 63:412–416. 10.1055/s-2001-15685 11467225 10.1055/s-2001-15685

[47] Chrubasik C, Roufogalis BD, Muller-Ladner U, Chrubasik S (2008) A systematic review on the Rosa canina effect and efficacy profiles. Phytother Res 22:725–733. 10.1002/ptr.2400 18384191 10.1002/ptr.2400

[48] Jäger AK, Eldeen IM, van Staden J (2007) COX‑1 and -2 activity of rose hip. Phytother Res 21:1251–1252. 10.1002/ptr.2236 17639563 10.1002/ptr.2236

[49] Yeşilada E, Ustün O, Sezik E et al (1997) Inhibitory effects of Turkish folk remedies on inflammatory cytokines: interleukin-1alpha, interleukin-1beta and tumor necrosis factor alpha. J Ethnopharmacol 9(7):76–77. 10.1016/s0378-8741 10.1016/s0378-8741(97)00076-7 9324006

[50] Kirkeskov B, Christensen R, Bügel S et al (2011) The effects of rose hip (Rosa canina) on plasma antioxidative activity and C‑reactive protein in patients with rheumatoid arthritis and normal controls: a prospective cohort study. Phytomedicine 18:953–958. 10.1016/j.phymed.2011.02.008 21420288 10.1016/j.phymed.2011.02.008

[51] Willich SN, Rossnagel K, Roll S et al (2010) Rose hip herbal remedy in patients with rheumatoid arthritis—a randomised controlled trial. Phytomedicine 17:87–93. 10.1016/j.phymed.2009.09.003 19818588 10.1016/j.phymed.2009.09.003

[52] Moré M, Gruenwald J, Pohl U, Uebelhack R (2017) A Rosa canina—Urtica dioica—Harpagophytum procumbens/zeyheri Combination Significantly Reduces Gonarthritis Symptoms in a Randomized, Placebo-Controlled Double-Blind Study. Planta Med 83:1384–1391. 10.1055/s-0043-112750 28614869 10.1055/s-0043-112750

[53] Winther K, Apel K, Thamsborg G (2005) A powder made from seeds and shells of a rose-hip subspecies (Rosa canina) reduces symptoms of knee and hip osteoarthritis: a randomized, double-blind, placebo-controlled clinical trial. Scand J Rheumatol 34:302–308. 10.1080/03009740510018624 16195164 10.1080/03009740510018624

[54] Warholm O, Skaar S, Hedman E et al (2003) The Effects of a Standardized Herbal Remedy Made from a Subtype of Rosa canina in Patients with Osteoarthritis: A Double-Blind, Randomized, Placebo-Controlled Clinical Trial. Curr Ther Res Clin Exp 0(3):4–3. 10.1016/s0011-393x 10.1016/S0011-393X(03)00004-3 PMC4053021 24944354

[55] Rein E, Kharazmi A, Winther K (2004) A herbal remedy, Hyben Vital (stand. powder of a subspecies of Rosa canina fruits), reduces pain and improves general wellbeing in patients with osteoarthritis—a double-blind, placebo-controlled, randomised trial. Phytomedicine 11:383–391. 10.1016/j.phymed.2004.01.001 15330493 10.1016/j.phymed.2004.01.001

[56] Christensen R, Bartels EM, Altman RD et al (2008) Does the hip powder of Rosa canina (rosehip) reduce pain in osteoarthritis patients?—a meta-analysis of randomized controlled trials. Osteoarthritis Cartilage 16:965–972. 10.1016/j.joca.2008.03.001 18407528 10.1016/j.joca.2008.03.001

[57] Goncalves C, Fernandes D, Silva I, Mateus V (2022) Potential Anti-Inflammatory Effect of Rosmarinus officinalis in Preclinical In Vivo Models of Inflammation. Molecules. 10.3390/molecules27030609 35163873 10.3390/molecules27030609 PMC8840442

[58] Habtemariam S (2023) Anti-Inflammatory Therapeutic Mechanisms of Natural Products: Insight from Rosemary Diterpenes, Carnosic Acid and Carnosol. Biomedicines. 10.3390/biomedicines11020545 36831081 10.3390/biomedicines11020545 PMC9953345

[59] Li L, Pan Z, Ning D, Fu Y (2021) Rosmanol and Carnosol Synergistically Alleviate Rheumatoid Arthritis through Inhibiting TLR4/NF-κB/MAPK Pathway. Molecules. 10.3390/molecules27010078 35011304 10.3390/molecules27010078 PMC8746366

[60] Liu M, Zhou X, Zhou L et al (2018) Carnosic acid inhibits inflammation response and joint destruction on osteoclasts, fibroblast-like synoviocytes, and collagen-induced arthritis rats. J Cell Physiol 233:6291–6303. 10.1002/jcp.26517 29521424 10.1002/jcp.26517

[61] von Schonfeld C, Huber R, Trittler R et al (2018) Rosemary has immunosuppressant activity mediated through the STAT3 pathway. Complement Ther Med 40:165–170. 10.1016/j.ctim.2018.03.004 30219443 10.1016/j.ctim.2018.03.004

[62] Khosravi MH, Atefi A, Mehri A et al (2023) Therapeutic effects of Rosa canina, Urtica dioica and Tanacetum vulgare herbal combination in treatment of tinnitus symptoms: A double-blind randomised clinical trial. Clin Otolaryngol 48:151–157. 10.1111/coa.13989 36268807 10.1111/coa.13989

[63] Lukaczer D, Darland G, Tripp M et al (2005) A pilot trial evaluating Meta050, a proprietary combination of reduced iso-alpha acids, rosemary extract and oleanolic acid in patients with arthritis and fibromyalgia. Phytother Res 19:864–869. 10.1002/ptr.1709 16261517 10.1002/ptr.1709

[64] Li L, Zhang H, Jin S, Liu C (2018) Effects of crocin on inflammatory activities in human fibroblast-like synoviocytes and collagen-induced arthritis in mice. Immunol Res 66:406–413. 10.1007/s12026-018-8999-2 29777367 10.1007/s12026-018-8999-2

[65] Ding Q, Zhong H, Qi Y et al (2013) Anti-arthritic effects of crocin in interleukin-1beta-treated articular chondrocytes and cartilage in a rabbit osteoarthritic model. Inflammation research : official journal of the European Histamine Research Society. Et Al 62:17–25. 10.1007/s00011-012-0546-3 10.1007/s00011-012-0546-3 22903188

[66] Tsiogkas SG, Grammatikopoulou MG, Gkiouras K et al (2021) Effect of Crocus sativus (Saffron) Intake on Top of Standard Treatment, on Disease Outcomes and Comorbidities in Patients with Rheumatic Diseases: Synthesis without Meta-Analysis (SWiM) and Level of Adherence to the CONSORT Statement for Randomized Controlled Trials Delivering Herbal Medicine Interventions. Nutrients. 10.3390/nu13124274 34959826 10.3390/nu13124274 PMC8706139

[67] Sahebari M, Heidari H, Nabavi S et al (2021) A double-blind placebo-controlled randomized trial of oral saffron in the treatment of rheumatoid arthritis. Avicenna J Phytomed 11:332–342. 10.22038/AJP.2020.17280 34290965 10.22038/AJP.2020.17280 PMC8264227

[68] Hamidi Z, Aryaeian N, Abolghasemi J et al (2020) The effect of saffron supplement on clinical outcomes and metabolic profiles in patients with active rheumatoid arthritis: A randomized, double-blind, placebo-controlled clinical trial. Phytother Res 34:1650–1658. 10.1002/ptr.6633 32048365 10.1002/ptr.6633

[69] Shakiba M, Moazen-Zadeh E, Noorbala AA et al (2018) Saffron (Crocus sativus) versus duloxetine for treatment of patients with fibromyalgia: A randomized double-blind clinical trial. Avicenna J Phytomed 8:513–523 30456199 PMC6235666

[70] Khayyal MT, El-Ghazaly MA, Abdallah DM et al (2005) Mechanisms involved in the anti-inflammatory effect of a standardized willow bark extract. Arzneimittelforschung 55:677–687. 10.1055/s-0031-1296917 16366042 10.1055/s-0031-1296917

[71] Fiebich BL, Chrubasik S (2004) Effects of an ethanolic salix extract on the release of selected inflammatory mediators in vitro. Phytomedicine 11:135–138. 10.1078/0944-7113-00338 15070163 10.1078/0944-7113-00338

[72] Kuppusamy UR, Khoo HE, Das NP (1990) Structure-activity studies of flavonoids as inhibitors of hyaluronidase. Biochem Pharmacol 40:397–401. 10.1016/0006-2952(90)90709-t 2375774 10.1016/0006-2952(90)90709-t

[73] Antoniadou K, Herz C, Le NPK et al (2021) Identification of Salicylates in Willow Bark (Salix Cortex) for Targeting Peripheral Inflammation. Int J Mol Sci. 10.3390/ijms222011138 34681798 10.3390/ijms222011138 PMC8540557

[74] Schmid B, Ludtke R, Selbmann HK et al (2000) Effectiveness and tolerance of standardized willow bark extract in arthrosis patients. Randomized, placebo controlled double-blind study. Z Rheumatol 59:314–320. 10.1007/s003930070053 11142926 10.1007/s003930070053

[75] Biegert C, Wagner I, Ludtke R et al (2004) Efficacy and safety of willow bark extract in the treatment of osteoarthritis and rheumatoid arthritis: results of 2 randomized double-blind controlled trials. J Rheumatol 31:2121–2130 15517622

[76] Beer AM, Wegener T (2008) Willow bark extract (Salicis cortex) for gonarthrosis and coxarthrosis—results of a cohort study with a control group. Phytomedicine 15:907–913. 10.1016/j.phymed.2008.07.010 18815018 10.1016/j.phymed.2008.07.010

[77] Oltean H, Robbins C, van Tulder MW et al (2014) Herbal medicine for low-back pain. Cochrane Database Syst Rev 2014:CD4504. 10.1002/14651858.CD004504.pub4 25536022 10.1002/14651858.CD004504.pub4 PMC7197042

[78] Uehleke B, Muller J, Stange R et al (2013) Willow bark extract STW 33‑I in the long-term treatment of outpatients with rheumatic pain mainly osteoarthritis or back pain. Phytomedicine 20:980–984. 10.1016/j.phymed.2013.03.023 23731658 10.1016/j.phymed.2013.03.023

[79] Federal Institute for Risk GA, Unit of Food Toxicology G, Matyjaszczyk E, Schumann R (2018) Risk assessment of white willow (Salix alba) in food. Efsa J 16:e16081. 10.2903/j.efsa.2018.e16081 32704312 10.2903/j.efsa.2018.e16081 PMC7374975

[80] Oketch-Rabah HA, Marles RJ, Jordan SA, Low Dog T (2019) United States Pharmacopeia Safety Review of Willow Bark. Planta Med 85:1192–1202. 10.1055/a-1007-5206 31604354 10.1055/a-1007-5206

[81] Geller AI, Shehab N, Weidle NJ et al (2015) Emergency Department Visits for Adverse Events Related to Dietary Supplements. N Engl J Med 373:1531–1540. 10.1056/NEJMsa1504267 26465986 10.1056/NEJMsa1504267 PMC6196363

[82] Schwarz S, Frolich L, Striebel JP, Hennerici MG (2007) The 12th German Drug Law (AMG) amendment: an obstruction for non-commercial clinical trials? Dtsch Med Wochenschr 132:108–112. 10.1055/s-2007-959298 17219346 10.1055/s-2007-959298

